# Chemical composition of four essential oils and their adulticidal, repellence, and field oviposition deterrence activities against *Culex pipiens* L. (Diptera: Culicidae)

**DOI:** 10.1007/s00436-024-08118-z

**Published:** 2024-01-25

**Authors:** Shaimaa M. Farag, Moataz A. M. Moustafa, Adrien Fónagy, Omnia M. H. M. Kamel, Doaa R. Abdel-Haleem

**Affiliations:** 1 Department of Entomology, Faculty of Science, Ain Shams University, Cairo, 11566 Egypt; 2 Department of Economic Entomology and Pesticides, Faculty of Agriculture, Cairo University, Giza, 12613 Egypt; 3 Zoology Department, Plant Protection Institute, Centre for Agricultural Research, ELKH (Eötvös Lóránd Research Network), 1022 Budapest, Hungary; 4 Applied Organic Chemistry Department, Institute of Industrial Chemistry Research, National Research Center, Giza, 12622 Egypt

**Keywords:** Adulticidal efficiency, Repellent, Oviposition deterrent, Essential oils, *Culex pipiens*

## Abstract

Effective mosquito repellents can limit the transmission of vector-borne diseases to humans. Consequently, there is an urgent need to develop mosquito control strategies that prioritize eco-friendly and cost-effective repellents. Essential oils (EOs) have enormous potential for mosquito repellency. Here, cinnamon, basil, eucalyptus, and peppermint EOs were investigated for adulticide and repellency properties against *Culex pipiens* as well on the oviposition behavior of gravid females from laboratory (lab test) and field (field test) populations. Cinnamon oil was an effective oviposition deterrent regardless of the population and had high adulticidal activity with toxicity index of 75.00% at 24 h of exposure, relative to deltamethrin. In addition, it exhibited effective repellency at 98.01% and 71.22% at 6.67 and 1.71 µl/cm^2^, respectively. Peppermint oil had the least adulticidal activity with toxicity index of 6.2% at 24 h, and it resulted in low repellency at 70.90% and 50.64% at 6.67 and 1.71 µl/cm^2^, respectively. On average, basil and eucalyptus oils showed some adulticidal efficiency, repellency, and oviposition deterrent activity. For all treatments, the oviposition deterrent index values of gravid females from natural populations (field test) were lower than those from lab-reared (lab test) females. Different ratios of monoterpenoids, phenylpropanoids, and fatty acids in the EOs tested likely account for the activity variations observed. Our results suggest cinnamon, basil, eucalyptus, and peppermint EOs, which are widely available, economical, and eco-friendly, with good potential for mosquito control strategies.

**Supplementary Information:**

The online version contains supplementary material available at 10.1007/s00436-024-08118-z.

## Introduction

The Culicidae, which are widely distributed in tropical regions of Africa, Asia, and Europe as well as the central regions of the Americas and Australia, are drivers of significant socioeconomic disruption (Vinogradova 2000). As one of the most devastating vector species in the world, *Culex pipiens* L. has been linked to the transmission of diverse human and animal diseases that cause millions of deaths annually (Lemine et al. 2017). Among the diseases and viruses vectored by *C. pipiens*, West Nile virus, St. Louis encephalitis, lymphatic filariasis, Rift Valley fever, and Sindbis are endemic and form epidemic areas in many countries (Turell 2012; Vloet et al. 2017; Ferraguti et al. 2021). The causative agents of diseases (i.e., virus, parasite) are transmitted to a host via the invasive feeding mechanism employed by female mosquitoes. Because of the low availability of vaccines, vector control remains the most effective method of disease prevention (WHO 2008). *Culex pipiens* is anthropophilic and inhabits natural sites in peri-domestic environments and frequently uses artificial containers (e.g., open drains, plant pots, buckets, water tanks, rain barrels, and other household containers) near human dwellings as oviposition sites (Njoroge and Berenbaum 2019). Recently, due to severe climatic changes that have led to the proliferation of mosquito oviposition and breeding sites, there have sharp increases in mosquito developmental and hatch rates that have contributed to a rise in mosquito populations and a concomitant amplification of mosquito-borne diseases (Deichstetter 2017).

Chemical insecticides play a vital role in vector control (Salem et al. 2023). The extended and widespread use of these chemicals for long-term public health applications, however, enhances the development of vector resistance and raises chemical pollution levels (Abbas et al. 2019; Ser and Cetin 2019). Developing alternative strategies for the control of adult mosquitoes necessitates exploring eco-friendly control methods. The use of mosquito repellents to protect human hosts and insecticides that reduce the mosquito population is crucial strategies for preventing vector-borne diseases (Manh and Tuyet 2020). Essential oils (EOs) are volatile, aromatic liquids produced from plant material by steam distillation (El-Shourbagy et al. 2023). They are composed of a mixture of highly volatile and lipophilic components including sesquiterpenes, phenols, coumarins, monoterpenes, anthraquinones, and alkaloids (Rios 2016; Sharifi-Rad et al. 2017). Many factors affect the chemical composition of EOs such as plant species and subspecies, part of the plant used, harvest time, geographical location, and the extraction methods used (Andrade-Ochoa et al. 2018). EOs are widely used in diverse commercial industries for numerous applications (*e.g.*, perfumes and cosmetics) and, due to their antioxidant and antimicrobial properties, are frequently sought for medicinal and pharmaceutical applications (Rios 2016). In addition, they also have applications as insect repellents and/or insecticides that can disrupt insect behavior, physiology, and biochemistry as well as induce neurotoxic effects (Krzyżowski et al. 2020). The EOs have been shown to have adulticide, larvicide, deterrence, and repellence activities against mosquitoes (Andrade-Ochoa et al. 2018; de Souza et al. 2019). Furthermore, EOs are effective, renewable, biodegradable, non-persistent in the environment, and relatively safe for non-target organisms and humans (Jalali Sendi and Ebadollahi 2014). Consequently, there is a strong demand to further develop EOs for mosquito control. The present study sought to investigate the adulticidal, repellence, and oviposition deterrence activities of EOs derived from *Cinnamomum verum* (cinnamon), *Ocimum basilicum* (basil), *Eucalyptus globulus* (eucalyptus), and *Mentha piperita* (peppermint) for adult *C. pipiens*.

## Materials and methods

### Plant oils

Four commercial essential oils (Table 1) were obtained from the National Research Center, Dokki, Giza, Egypt, and EL CAPTAIN® Company for extracting natural oils, “Cap Pharm,” El Obor, Cairo, Egypt.

**Table 1:** Names and taxonomic classification of the essential oils (EOs)

No	Oil name	Scientific name	Order	Family
1	Cinnamon	*Cinnamomum verum*	Laurales	Lauraceae
2	Basil	*Ocimum basilicum*	Lamiales	Lamiaceae
3	Tasmanian blue gum	*Eucalyptus globulus*	Myrtales	Myrtaceae
4	Peppermint	*Mentha piperita*	Lamiales	Lamiaceae

### Gas chromatography–mass spectrometry (GC–MS) analysis

The chemical composition of *C. verum*, *O. basilicum*, *E. globulus*, and *M. piperita* EOs was identified using a Shimadzu single quadrupole gas chromatograph–mass spectrometer (GC–MS-QP) 2015 plus (Kyoto, Japan) via 0.5 µl injections of the respective EO on a Hewlett Packard chromatograph model 597 equipped with a flame ionization detector (FID) and a 50-cm HP capillary column. The oven temperature increased from 60 to 200 °C for 25 min at 3 °C/min. The injector and detector temperatures were 200 and 250 °C, respectively. The carrier gas was helium at a flow rate of 1 ml/min. Diluted samples (1v/v) were injected in a 10 µl volume with a 15:1 split ratio. The MS parameters were as follows: interface temperature 280 °C, ion source temperature 200 °C, electron ionization (EI) mode set at 70 Ev, and a 35–500 amu scan range. To identify the obtained peaks, the retention time (RT) of each peak was compared with that of the authentic; component quantities were determined by comparing peak areas with data in the WILEY/NIST and Tutor Libraries (Beckley et al. 2014; Abd El-Kareem et al. 2016).

### Maintenance of mosquito culture

The laboratory strain of *C. pipiens* L. was continuously maintained at the Research and Training Center for Vectors of Diseases (Faculty of Science, Ain Shams University, Egypt) for several generations at 70 ± 5% relative humidity, 27 ± 2 °C, and a 10:14 h (D:L) regime without previous exposure to insecticides (Kasap and Demirhan 1992; Abdel-Haleem et al. 2020).

### Adulticidal activity

The toxicity of the four tested EOs was evaluated against adult *C. pipiens* according to the WHO (2013) bioassay with some modifications. The stock solutions were prepared by dissolving EOs in ethanol (commercial 95%) and then diluting in the same solvent to obtain serial concentrations (0.02%, 0.05%, 0.1%, 0.5%, 1%, and 2%) of each oil. The inner surface of the WHO tube was coated with each concentration and left for 2 h to allow for ethanol evaporation. A batch of 20 4–5-day-old mixed-sex adults fed on 10% sugar solution was transferred to each tube by a hand aspirator. This process was repeated three times for each concentration.

After 1-h exposure, the mosquito groups were transferred to clean cubs (without tested materials) with 10% sucrose solution for recovery. Deltamethrin (deltamethrin 98% technical; Rudong Zhongyi Chemical Co., Ltd, Rudong, Jiangsu Province, China) was obtained from the Central Agricultural Pesticide Laboratory (Dokki, Giza, Egypt) and used as a positive control at the WHO recommended concentration (0.05%). The solvent control consisted of tubes prepared with ethanol alone. Mortality was recorded 6-, 12-, and 24-h post-exposure. The corrected mortality percentages were estimated according to Abbott’s formula (Abbott 1925).

$$\mathrm{\%\;corrected\;mortality}=[(\mathrm{\%\;test\;kill}\hspace{0.17em}-\hspace{0.17em}\mathrm{\%\;control\;kill})/(100\hspace{0.17em}-\hspace{0.17em}\mathrm{\%\;control\;kill})]\hspace{0.17em}\times \hspace{0.17em}100$$

### Repellent activity

Standard cages (25 × 25 × 25 cm^3^) were used to evaluate the repellence of the EOs for *C. pipiens* females and 15% N,N-diethyl-meta-toluamide (DEET) in a commercial brand (Off®; Johnson Wax, Giza Egypt) was used as a positive control. Four different concentrations (6.67, 3.33, 2.57, and 1.71 µl/cm^2^) of each EO were prepared by dissolving each concentration in 2 ml ethanol with a small drop (10 µl) of Triton X100. Controls consisted of ethanol alone with a drop of Triton X100. A 0.5 µl aliquot of each EO concentration or control (using micropipette) was directly applied onto a 5 × 5 cm^2^ region of a pigeon abdomen devoid of feathers. After 10 min, pigeons were placed for 4 h in cages containing previously starved *C. pipiens* females (laboratory strain). The unfed females were counted. Each treatment was repeated three times and the mean repellent activity value was determined (El-Sheikh et al. 2016; El Hadidy et al. 2022). The repellency was recorded and analyzed according to the Abbott formula, (Abbott 1925):

$$\mathrm{The\;repellency\;\%}=\left(\mathrm{A\%}-\mathrm{B\%}/100-\mathrm{B\%}\right)\times 100$$

where A% is the percentage of unfed females in treatment; B% is the percentage of unfed females in control.

### Laboratory oviposition deterrence activity

To evaluate the effects of the EOs on the oviposition behavior of gravid C*. pipiens* females, deterrence assays were performed according to Njoroge and Berenbaum (2019). Newly mated females were fed 10% sucrose and blood-fed on a pigeon. Six different concentrations (0.1, 0.5, 1, 2, 4, and 6%) of each EO in 100 ml water mixed with a drop of Triton X100 were prepared and aliquoted into disposable cups (150 ml). For each concentration, ten gravid female mosquitoes (fed a single blood meal) were placed in a wooden cage (25 × 25 × 25 cm^3^) containing five treatment oviposition cups and the control cup. Three replicates were used for each concentration. The control consisted of water and Triton X100 only. In addition, a 10% sucrose solution diet was provided in each cage. Conditions for each of the tested EOs were the same as rearing. The number of eggs was counted under a stereomicroscope at 5-day post-treatment.

### Field oviposition deterrence activity

The field oviposition deterrent test was performed in the rural area at El Nazlah (29° 18ʹ 54.6″ N, 30° 38ʹ 33.6″ E; Yossef Elsedik district, El Fayoum Governorate, Egypt). To evaluate the oviposition deterrence of the EOs against *C. pipiens* under field conditions, the six concentrations described above were prepared in 3 L of water and added to a plastic container (5 L capacity, 25 cm diameter, 30 cm high). A section of white filter paper (20 cm × 10 cm wide) placed at the bottom of each container but in contact with the water surface served as an oviposition surface. Three replicate containers of each concentration were placed randomly under selected trees as a shelter and inspected daily. The ovistrip filter paper was removed from the containers after 1 week and the number of eggs was determined under a stereomicroscope. The oviposition deterrence results are presented as a mean number of laid eggs and the oviposition activity index (OAI), which was estimated according to the following formula (Kramer and Mulla 1979):

$$OAI=\left({N}_{t}-{N}_{s}\right)/{N}_{t}+{N}_{s}$$

where *N*_t_ is the total number of eggs in the test treatment and *N*_s_ is the total number of eggs in the control.

The range of the oviposition activity indices (OAI) lies from + 1 to − 1. EOs with positive values are considered attractants (more eggs were deposited in the treatment cups than in the control cups), while those with negative values are considered repellents (more eggs were deposited in the control cups than in the treatment cups) (Prathibha et al. 2014).

### Statistical analysis

LC_50_ and LC_90_ values of the tested EOs were calculated using LdPLine©) software with the Log-Probit analysis method (Finney 1971). Adulticidal toxicity indices for the EOs were estimated according to (Sun 1950). The repellent and oviposition deterrent parameters were analyzed by one-way analysis of variance (ANOVA) using IBM SPSS Statistics v 19.0. Estimates of EO concentration mean differences were conducted depending on the significance level (*P* ≤ 0.05) using Tukey’s HSD test. *C. pipiens* mortality curves in response to the tested EOs were generated using Graph Pad Prism v 9.

## Results

### Gas chromatography–mass spectrophotometry (GC–MS) analysis of the tested essential oils

**Fig. 1:**
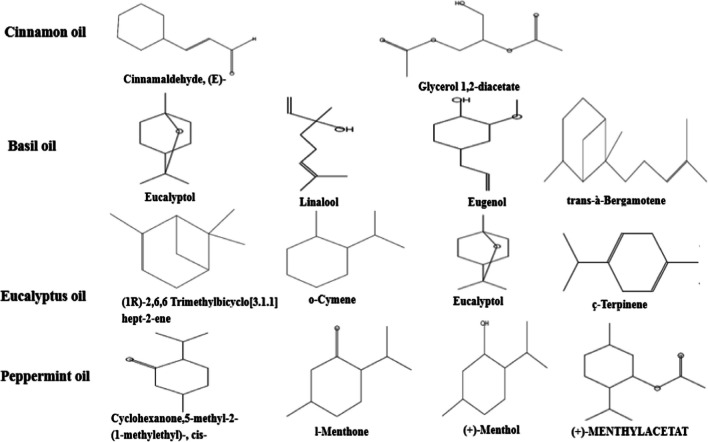
Chemical structure of the main bioactive compounds in cinnamon (*Cinnamomum verum*), basil (*Ocimum basilicum*), Tasmanian blue gum (*Eucalyptus globulus*), and peppermint (*Mentha piperita*) essential oils

GC–MS analysis revealed the four EOs (cinnamon, basil, eucalyptus, and peppermint) contained differing amounts of various bio-active components (Table 2). The chemical component, retention time (RT), percent peak area (*i.e.*, average concentration), molecular weight, and molecular formula of the compounds identified in the EOs are shown in Table 2. The chemical structure of the principle components in the respective EOs is shown in Fig. 1. Cinnamon oil was composed mainly of three components that accounted for 100% of the total composition: cinnamaldehyde (67.59%), glycerol 1,2-diacetate (29.03%), and phenol,2-methoxy-4-(2-propenyl) (2.68%) (Table 2). The major components in basil EO were largely monoterpenes, represented by linalool (20.07%), trans-α-bergamotene (10.63%), eucalyptol (8.80%), and eugenol (8.62%). Eucalyptus EO was similarly rich in monoterpenes (Table 2), which accounted for 79.63% of the compounds and included eucalyptol (49.34%), o-cymene (17.78%), and ç-terpinene (12.51%). In addition, small traces of (2,6,6-trimethylbicyclo[3.1.1]hept-2-ene) were detected. The predominant compounds in peppermint EO were monoterpenoids (Table 2), including menthol (34.09%), l-menthone (10.73%), ( +)-menthylacetat (9.48%), and levomenthol (4.90%). Small traces of the monoterpenes eucalyptol (6.97%) and isopulegol (1.67%) were also present.

**Table 2:** Chemical composition of essential oils from cinnamon (*Cinnamomum verum*), basil (*Ocimum basilicum*), Tasmanian blue gum (*Eucalyptus globulus*), and peppermint (*Mentha piperita*)

No	RT	Compound name	Area %	Molecular formula	Molecular weight
Cinnamon oil (*Cinnamomum verum*)					
1	11.53	(E)-Cinnamaldehyde	68.29	C_9_H_8_O	132
3	13.34	Glycerol 1,2-diacetate	29.03	C_7_H_12_O_5_	176
4	13.48	Phenol,2-methoxy-4-(2-propenyl)	2.69	C_10_H_12_O_2_	164
Basil oil (*Ocimum basilicum*)					
6	3.66	1,3,7-Octatriene,3,7-dimethyl	0.71	C_10_H_16_	136
7	3.93	Camphene	0.15	C_10_H_16_	136
8	4.29	Bicyclo[3.1.0]hexane,4-methylene-1-(1 methylethyl)	0.57	C_10_H_16_	136
9	4.39	Bicyclo[3.1.1]heptane,6,6-dimethyl-2 methylene-, (1S)-	1.18	C_10_H_16_	136
10	4.56	á-Myrcene	0.72	C_10_H_16_	136
11	5.28	p-Cymene	0.30	C_10_H_14_	134
12	5.45	Eucalyptol	8.80	C_10_H_18_O	154
13	5.73	á-Ocimene	0.23	C_10_H_16_	136
14	6.30	Cyclohexanol,1-methyl-4-(1 methylethenyl)-, cis-	0.33	C_10_H_18_O	154
15	6.65	2- Furanmethanol,5-ethenyltetrahydro-à,à,5-trimethyl-,cis	0.22	C_10_H_18_O_2_	170
16	7.04	Linalool	20.07	C_10_H_18_O	154
17	7.90	Cis-epoxy-ocimene	0.20	C_10_H_16_O	152
18	8.10	Camphor	0.79	C_10_H_16_O	152
19	8.54	l-Menthone	0.14	C_10_H_18_O	154
20	8.76	Bicyclo[2.2.1]heptan-2-OL,1,7,7-trimethyl	0.49	C_10_H_18_O	154
21	8.97	Terpinen-4-ol	0.69	C_10_H_18_O	154
22	9.40	Estragole	2.70	C_10_H_12_O	148
23	9.70	5-Isopropenyl-2-methyl-2-cyclohexan-1-OL	0.26	C_10_H_16_O	152
24	10.18	6-Octen-1-OL, 3,7-dimethyl-	0.70	C_10_H_20_O	156
25	10.80	2,6-Octadien-1-OL,3,7-Dimethyl-, (Z)-	0.21	C_10_H_18_O	154
26	11.32	6-Octen-1-ol, 3,7-dimethyl-, formate	0.14	C_11_H_20_O_2_	184
27	11.58	Acetic acid,1,7,7-trimethyl bicyclo[2.2.1]hept-2 ester	1.89	C_12_H_20_O_2_	196
28	12.97	2-Oxabicyclo[2.2.2]octan-6-ol,1,3,3-trimethyl-, acetate	0.18	C_12_H_20_O_3_	212
29	13.20	à-Cubebene	0.15	C_15_H_24_	204
30	13.41	Eugenol	8.62	C_10_H_12_O_2_	164
31	13.93	Tricyclo[4.4.0.0(2,7)]DEC-3-ene,1,3-dimethyl-8-(1-methylethyl)-	0.37	C_15_H_24_	204
32	14.13	( −)-á-Bourbonene	0.54	C_15_H_24_	204
33	14.30	Cyclohexane,1-ethenyl-1-methyl-2,4-bis(1-methylethenyl)-, [1S-(1à,2á,4á)]-	4.77	C_15_H_24_	204
34	14.40	Isoledene	0.18	C_15_H_24_	204
35	14.60	Methyleugenol	0.20	C_11_H_14_O_2_	178
36	14.87	Cis-à-bergamotene	0.14	C_15_H_24_	204
37	15.01	Caryophyllene	0.52	C_15_H_24_	204
38	15.12	Cedrene	0.12	C_15_H_24_	204
39	15.27	1H-Cyclopropa[a]naphthalene,1a,2,3,5,6,7,7a,7b-octahydro-1,1,7,7a-tetramethyl-	0.15	C_15_H_24_	204
40	15.38	Trans-à-Bergamotene	10.63	C_15_H_24_	204
41	15.55	Caryophyllene	0.11	C_15_H_24_	204
42	15.89	Humulene	1.62	C_15_H_24_	204
43	16.06	1,6-Cyclodecadiene,1-methyl-5-methylene-8-(1-methylethyl)-	6.38	C_15_H_24_	204
44	16.37	Alloaromadendrene	0.20	C_15_H_24_	204
46	16.70	Naphthalene,decahydro-4a-methyl-1-methylene-7-(1-methylethenyl)-	0.26	C_15_H_24_	204
47	16.87	Azulene,1,2,3,3a,4,5,6,7-octahydro-1,4-dimethyl-7-(1-methylethenyl)-	4.35	C_15_H_24_	204
49	17.14	Germacrene A	0.93	C_15_H_24_	204
50	17.30	ç-Muurolene	5.81	C_15_H_24_	204
51	17.45	4-Isopropyl-1,6-dimethyl-1,2,3,4 tetrahydronaphthalene	1.35	C_15_H_22_	202
52	17.55	( +)-á-Funebrene	0.35	C_15_H_24_	204
53	17.71	(3S,3aR,3bR,4S,7R,7aR)-4-Isopropyl-3,7-dimethyl octahydro-1H cyclopenta [1,3] cyclopropa [1,2] benzen-3-ol	0.11	C_15_H_26_O	222
54	17.85	à-Muurolene	0.12	C_15_H_24_	204
55	18.31	Caryophylla-4(12),8(13)-dien-5à-ol	0.16	C_15_H_24_O	220
56	18.46	Nerolidol	0.17	C_15_H_26_O	222
57	18.60	(1aR,3aS,7S,7aS,7bR)-1,1,3a,7-Tetramethyl decahydro-1H-cyclopropa[a] naphthalene 7-ol	0.28	C_15_H_26_O	222
58	18.80	( −)-Spathulenol	1.07	C_15_H_24_O	220
59	19.51	( −)-5-Oxatricyclo[8.2.0.0(4,6)]dodecane,12-trimethyl-9-methylene-,[1R-(1R*,4R*,6R*,10S*)]	0.12	C_15_H_24_O	220
60	19.68	Epicubenol	1.34	C_15_H_26_O	222
61	19.81	2Naphthalenemethanol,1,2,3,4,4a,5,6,7octahydroà,à4a,8-tetramethyl-, (2R-cis)-	0.39	C_15_H_26_O	222
62	20.09	( −)-Spathulenol	0.15	C_15_H_24_O	220
63	20.30	Tau.-Cadinol	5.83	C_15_H_26_O	222
64	20.58	Tau.-Muurolol	0.27	C_15_H_26_O	222
65	20.64	Alloaromadendrenoxixid-(1)	0.25	C_15_H_24_O	220
66	27.45	D-glucose, 5TMS derivative	0.12	C_21_H_52_O_6_Si_5_	540
67	28.58	Palmitic acid, TMS derivative	0.18	C_19_H_40_O_2_Si	328
Tasmanian blue gum oil (*Eucalyptus globulus*)					
68	3.64	(1R)-2,6,6-Trimethylbicyclo[3.1.1]hept-2-ene	13.18	C_10_H_16_	136
69	4.38	Bicyclo[3.1.1]heptane,6,6-dimethyl-2-methylene	1.06	C_10_H_16_	136
70	4.55	á-Myrcene	1.25	C_10_H_16_	136
71	4.90	1,3-Cyclohexadiene,2-methyl-5-(1-methylethyl)-	2.08	C_10_H_16_	136
72	5.12	Cyclohexene,1-methyl-4-(1-methylethylidene)-	0.51	C_10_H_16_	136
73	5.30	o-Cymene	17.78	C_10_H_14_	134
74	5.44	Eucalyptol	49.34	C_10_H_18_O	154
75	6.00	ç-Terpinene	12.51	C_10_H_16_	136
76	6. 62	Cyclohexene,1-methyl-4-(1-methylethylidene)-	0.51	C_10_H_16_	136
77	8.98	Terpinen-4-ol	0. 68	C_10_H_18_O	154
78	9.38	L-à-terpineol	1.11	C_10_H_18_O	154
Peppermint oil (*Mentha piperita*)					
79	3.66	3-Carene	1.21	C_10_H_16_	136
80	4.29	Bicyclo[3.1.0]hexane,4-methylene-1-(1-methylethyl)-	0.58	C_10_H_16_	136
82	4.56	Bicyclo[3.1.1]heptane,6,6-dimethyl-2-methylene	1.58	C_10_H_16_	136
84	5.29	o-Cymene	0.87	C_10_H_14_	134
85	5.38	Cyclohexene,1-methyl-4-(1-methylethenyl)-, (S)-	2.45	C_10_H_16_	136
86	5.45	Eucalyptol	6.97	C_10_H_18_O	154
87	6.01	ç-Terpinene	0.17	C_10_H_16_	136
88	6.98	Linalool	0.25	C_10_H_18_O	154
89	8.17	Isopulegol	1.67	C_10_H_18_O	154
90	8.36	Cyclohexanone,5-methyl-2-(1-methylethyl)-, cis	17.91	C_10_H_18_O	154
91	8.56	l-Menthone	10.73	C_10_H_18_O	154
92	8.73	Levomenthol	4.90	C_10_H_20_O	156
93	9.00	( +)-Menthol	34.09	C_10_H_20_O	156
94	9.22	Levomenthol	0.67	C_10_H_20_O	156
95	9.38	L-à-Terpineol	0.49	C_10_H_18_O	154
96	10.43	Cyclohexanone,5-methyl-2-(1-methylethylene)-, (R)-	3.19	C_10_H_16_O	152
97	10.83	2-Cyclohexen-1-one,3-methyl-6-(1-methylethyl)-	0.76	C_10_H_16_O	152
98	11.29	Cyclohexanol,5-methyl-2-(1-methylethyl)-, acetate	0.20	C_12_H_22_O_2_	198
99	11.76	( +)-Menthylacetat	9.48	C_12_H_22_O_2_	198
100	14.13	( −)-á-Bourbonene	0.26	C_15_H_24_	204
101	15.02	Caryophyllene	1.34	C_15_H_24_	204
102	16.52	1,6-Cyclodecadiene,1-methyl-5-methylene-8-(1-methylethyl)-, [S-(E,E)]-	0.17	C_15_H_24_	204
103	18.88	( −)-5-Oxatricyclo[8.2.0.0(4,6)] dodecane,12-trimethyl-9-methylene-,[1R (1R*,4R*,6R*,10S*)]	0.18	C_15_H_24_O	220

*RT* retention time (min)

### Adulticidal efficacy

**Fig. 2:**
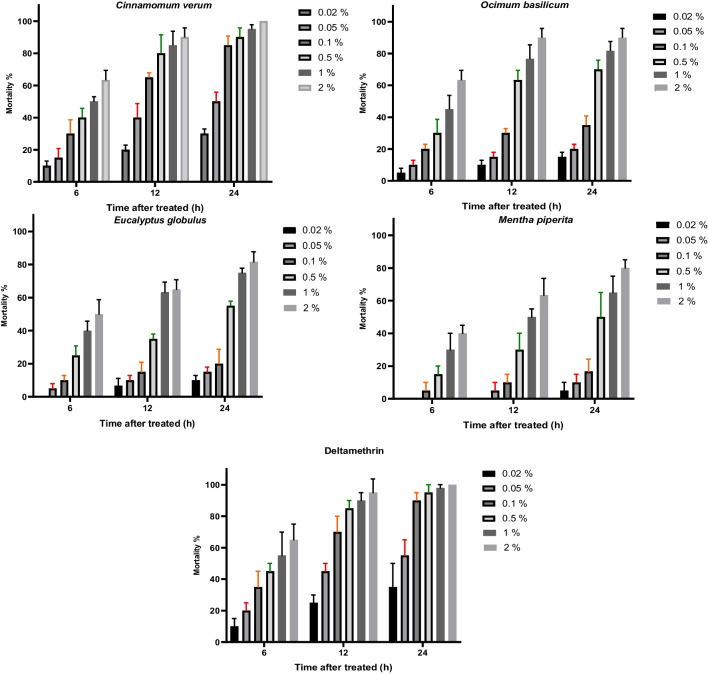
Cumulative mortality (mean ± SE) of *Culex pipiens* after 6-, 12-, and 24-h exposure to six different concentrations (0.02, 0.05, 0.1, 0.5, 1, and 2%) of the tested EOs and deltamethrin

The adulticide activity of the tested EOs on *C. pipiens* adults was compared relative to deltamethrin*.* Mortality was determined after exposure for 6, 12, and 24 h under laboratory conditions (Fig. 2). The adulticidal activity of the EOs increased gradually with exposure time and the highest mortality was observed at 24-h exposure. Cinnamon EO and deltamethrin exhibited the best efficiency (*F* = 4.25, *P* = 0.0032 and *F* = 16.24, *P* =  < 0.0001, respectively) at all exposure times relative to the other EOs (Fig. 2). In contrast, peppermint EO had the least adulticidal activity (*F* = 14.88, *P* =  < 0.0001). No mortality was observed in the control group. After 24-h exposure, the ranking of the EO LC_50_ values was as follows: cinnamon (0.04%) > basil (0.18%) > eucalyptus (0.33%) > peppermint (0.49%) (Table 3).

**Table 3:** Toxicity of tested essential oils and deltamethrin on *C. pipiens*. The tested EOs were applied (0.02, 0.05, 0.1, 0.5, 1, and 2%) to the inner surface of a WHO tube. A positive control of deltamethrin (0.05%) was similarly applied. *C. pipiens* adults were exposed for 1 h and then transferred to a clean tubes. LC values were calculated 24 h post-exposure. Treatments were performed in triplicate with each replicate consisting of 20 adults. LC_50_ and LC_90_ values of the tested EOs were calculated with LdPLine software according to the Log-Probit analysis method (Finney 1971)

Treatments	LC_50_ (95% confidence limits)	LC_90_ (95% confidence limits)	Slope ± SE^a^	*χ*^2b^	*P*^*c*^	Toxicity index
Cinnamon oil (*C. verum*)	0.04 (0.01–0.06)	0.37 (0.24–1.19)	1.32 ± 0.11	14.76	0.005	75.00
Bail oil (*O. basilicum*)	0.18 (0.14–0.22)	1.921 (1.36–2.94)	1.25 ± 0.08	2.92	0.57	16.60
Tasmanian blue gum oil (*E. globulus)*	0.33 (0.27–0.41)	3.63 (2.48–5.90)	1.23 ± 0.09	4.97	0.28	9.12
Peppermint oil (*M. piperita*)	0.49 (0.40–0.62)	4.79 (3.25–7.88)	1.30 ± 0.09	1.57	0.81	6.23
Deltamethrin	0.03 (0.01–0.07)	0.19 (0.09–0.59)	1.49 ± 0.13	13.68	0.008	100

^a^Slope of the concentration–inhibition regression line ± standard error

^b^(*χ*2) Chi square value

^c^(*P*) probability

The toxicity index (Sun 1950) was employed for the direct comparison of insecticides

Toxicity index (Sun’s equation) = LC_50_ of the most effective compound/LC_50_ of the tested compound × 100

### Repellence activity

The feeding deterrence effects of the EOs against *C. pipiens* females are shown in Table 4. The repellent efficacy gradually increased with the EO concentration as repellency was more effective at 6.67 µl/cm^2^ than at 1.71 µl/cm^2^. Cinnamon EO had the highest repellency (98.01%) at 6.67 µl/cm^2^, which was comparable to that of the DEET control at 100%. Basil and eucalyptus EOs had moderate repellence activities and peppermint EO had significantly lower potency (Table 4).

**Table 4:** Repellency of the tested EOs on female *C. pipiens.* The tested EOs were directly applied to the abdomen of pigeon for 10 min. Each EO was applied as 6.67, 3.33, 2.57 and 1.71%. After coating, each treated pigeon was placed for 4 h in cages containing starved *C. pipiens* females. Each treatment was repeated three times and the mean repellent activity value was determined

Essential oils	Dose (µl/cm^2)^	Number of tested females	% fed	% unfed	Repellency %
Control		55	91.86 ± 1.83^a^	8.14 ± 1.83^a^	-
Cinnamon oil (*C. verum*)	6.67	55	1.66 ± 1.66^d^	98.34 ± 1.66^d^	98.01
	3.33	46	10.83 ± 2.07^c^	89.17 ± 2.07^c^	88.16
	2.57	59	15.26 ± 0.26^c^	84.74 ± 0.26^c^	83.38
	1.71	53	26.36 ± 1.43^b^	73.64 ± 1.43^b^	71.22
Bail oil (*O. basilicum*)	6.67	61	14.76 ± 0.23^d^	85.24 ± 0.23^d^	84.68
	3.33	48	18.70 ± 0.00^c^	81.30 ± 0.00^d^	80.54
	2.57	42	26.13 ± 0.56^c^	73.87 ± 0.56^c^	72.82
	1.71	49	40.80 ± 0.40^b^	59.20 ± 0.40^b^	57.64
Tasmanian blue gum oil (*E. globulus*)	6.67	51	21.46 ± 0.73^e^	78.54 ± 0.73^e^	76.37
	3.33	48	27.53 ± 1.23^d^	72.46 ± 1.23^d^	70.33
	2.57	50	36.03 ± 0.73^c^	63.96 ± 0.73^c^	60.57
	1.71	53	47.06 ± 0.36^b^	52.93 ± 0.36^b^	48.34
Peppermint oil (*M. piperita*)	6.67	50	28.40 ± 0.26^d^	71.60 ± 0.30^d^	70.90
	3.33	46	34.63 ± 0.66^c^	65.37 ± 0.66^c^	63.85
	2.57	53	45.30 ± 0.90^b^	54.70 ± 0.90^b^	52.94
	1.71	40	47.63 ± 1.19^b^	52.37 ± 1.19^b^	50.64

Values followed by the same letters are not significantly different (Tukey’s HSD test, *P* < 0.05)

### Oviposition deterrence activity

The efficacy of the EOs in deterring oviposition behavior in both laboratory and field-based tests is summarized in Table 5. Although oviposition in the low EO groups (0.1 and 0.5%) differed from the control group under laboratory conditions, more significant deterrence effects were observed at the higher concentrations (Table 5). Deterrence effects are characterized by diminished egg-laying capacities and were most pronounced at 6% EO. In contrast, the 0.1% EO groups had weak oviposition deterrence effects (Table 5). Overall, cinnamon had the strongest effects followed by comparable effects from the basil and eucalyptus and then peppermint.

**Table 5:** Oviposition deterrence activity of the tested EOs against gravid female *Culex pipiens* under laboratory and field conditions. Ten gravid female mosquitoes were placed in a wooden cage (25 × 25 × 25 cm) containing oviposition cups treated with each of the respective EOs or control. Each EO was applied at six different concentrations (0.1, 0.5, 1, 2, 4, and 6%) with three replicates for each concentration. The total number of eggs laid was determined at 5-day post-treatment and the oviposition activity index (OAI) was calculated. The same concentrations were used for the field assays

Concentration (%)	Cinnamon oil (*C. verum*)		Basil oil (*O. basilicum*)		Tasmanian blue gum oil (*E. globulus*)		Peppermint oil (*M. piperita*)	
	No. of eggs	OAI	No. of eggs	OAI	No. of eggs	OAI	No. of eggs	OAI
Laboratory								
Control	2636.00 ± 5.19^a^	00	2717.00 ± 7.50^a^	00	1609.67 ± 691.34^a^	00	2395.00 ± 9.81^a^	00
0.1	1238.00 ± 1.15^b^	–0.36	1447.00 ± 4.04^b^	–0.30	1178.00 ± 4.61^ab^	–0.31	1836.00 ± 6.92^b^	–0.13
0.5	1011.00 ± 1.15^c^	–0.44	1015.00 ± 5.19^c^	–0.45	1095.00 ± 6.35^ab^	–0.35	1559.00 ± 5.19^c^	–0.21
1	735.00 ± 2.88^d^	–0.56	937.00 ± 3.46^d^	–0.48	764.00 ± 5.77^ab^	–0.50	1105.00 ± 6.35^d^	–0.36
2	685.00 ± 2.88^e^	–0.58	727.00 ± 4.04^e^	–0.57	617.00 ± 4.04^ab^	–0.57	584.00 ± 3.46^e^	–0.60
4	329.00 ± 3.46^f^	–0.77	468.00 ± 6.35^f^	–0.70	349.00 ± 5.19^ab^	–0.73	525.00 ± 4.04^f^	–0.64
6	106.00 ± 2.30^ g^	–0.92	102.00 ± 2.88^ g^	–0.92	226.00 ± 2.30^b^	–0.82	276.00 ± 4.04^ g^	–0.79
Field								
Control	2328.00 ± 38.08^a^	00	2292.00 ± 27.07^a^	00	2385.33 ± 73.97^a^	00	2086.00 ± 25.94^a^	00
0.1	1200.00 ± 27.71^b^	–0.32	1182.00 ± 24.007^b^	–0.32	1400.00 ± 22.89^b^	–0.27	1772.00 ± 17.05^b^	–0.08
0.5	1090.00 ± 19.63^b^	–0.36	1079.00 ± 18.35^c^	–0.36	1288.00 ± 15.94^b^	–0.31	1555.00 ± 20.59^c^	–0.15
1	933.00 ± 30.60^c^	–0.43	1043.00 ± 18.33^c^	–0.37	1055.00 ± 14.29^c^	–0.40	1200.00 ± 14.01^d^	–0.27
2	659.00 ± 19.65^d^	–0.56	752.00 ± 16.37^d^	–0.51	822.00 ± 9.29^d^	–0.50	912.00 ± 9.16^e^	–0.39
4	406.00 ± 17.57^e^	–0.70	470.00 ± 13.05^e^	–0.66	500.00 ± 16.50^e^	–0.66	565.00 ± 7.93^f^	–0.57
6	199.00 ± 13.45^f^	–0.84	223.00 ± 15.69^f^	–0.82	265.00 ± 14.01^f^	–0.80	282.00 ± 6.11^ g^	–0.76

Values followed by the same letters are not significantly different (Tukey’s HSD test, *P* < 0.05)

Under field conditions, significant effects on oviposition were observed with the cinnamon and basil EOs at multiple concentrations (0.1, 0.5, 1, and 2%) relative to the control (Table 5). In contrast, peppermint EO had the weakest effects with low oviposition deterrent indices − 0.08 and − 0.15 at 0.1 and 0.5%, respectively. Overall, the least effective oviposition deterrence was observed in the peppermint EO groups (Table 5). With respect to oviposition preference, cinnamon EO reduced the number of eggs laid by both laboratory-reared and field populations of *C. pipiens* and the basil and eucalyptus EO had similar oviposition activity indices. However, all four EOs tested displayed effective oviposition deterrence activities at high concentrations.

## Discussion

Mosquito-borne diseases are serious public health problems in most developing countries. The spread and incidence of these diseases, however, can be controlled by using adulticidal agents or repellents that limit mosquito feeding and oviposition (Prathibha et al. 2014). Chemicals extracted from plants can have repellence, feeding deterrence, toxic, and growth regulation effects. Although the main function of these plant chemicals may be defensive against phytophagous insects, many volatile components are also effective repellents against hematophagous insects such as mosquitoes (Maia and Moore 2011). In addition, the use of natural products like EOs is advantageous due to their environmental friendliness, compatibility, and degradability (Vatandoost et al. 2008). Several EOs have been widely recommended as mosquito repellents (Maia and Moore 2011). Mosquitoes locate their hosts by olfactory, visual, and thermal cues. Mosquitoes detect human host odors like acid lactic, CO_2_, and 1-octen-3-ol via odorant receptor sites typically housed in their antenna (Raji and DeGennaro 2017). It has been suggested that mosquito repellent modes of action may be based on the inhibition of receptors associated with attraction or the activation of receptors associated with repellency (Dickens and Bohbot 2013). Thus, EOs that disrupt odorant receptor interactions can reduce contact between mosquitoes and their human hosts (Barnard 1999; Manh and Tuyet 2020). However, several EOs have a variety of neurotoxic mechanisms of action, such as inhibition of acetylcholinesterase (Houghton et al. 2006) and glutathione S-transferase (Moustafa et al. 2023), disruption of GABA (Priestley et al. 2003), or disruption of octopamine receptors (Enan 2001). High monoterpene extracts (EOs) usually influence GABA, tyramine, and octopamine receptors in addition to TRP channels (Ferreira et al. 2019). Oviposition prevention could result from adult mosquitoes undergoing behavioral and physiological modifications that negatively impact their ability to deposit eggs. It has been demonstrated that some phytochemicals function as growth inhibitors, interfering with either reproduction or development and growth (Rajkumar and Jebasan 2009).

Overall, EOs represent a complex range of secondary metabolites with deleterious effects on insects that can interact synergistically to enhance their effectiveness (Rossi and Palacios 2015; Tak and Isman 2015). A mixture of trans-anethole and thymol has increased potency against *Spodoptera litura* (Hummelbrunner and Isman 2001), and clove EO is more effective than its major component (eugenol) alone. Likewise, *Mentha arvensis* EO has higher *Aedes aegypti* larvae toxicity than menthol (major component). It has been suggested that minor compounds in the EO might synergize with the major constituents to improve toxicity (Santos et al. 2011; Osanloo et al. 2018).

In this study, widely available and economical EOs were assayed for adulticidal, oviposition deterrence, and repellence activities. In agreement with a study by Kowalska et al. (2021), which reported that the cinnamon EO is effective against many insect pests, we found that it had high repellency and adulticidal efficacy against adult *C. pipiens*. Similarly, cinnamon EO showed significant repellency against female and male *C. quinquefasciatus* adults (Nakasen et al. 2021). This effectiveness is likely due to the high bioactive compound content as cinnamaldehyde, a phenylpropanoid, is the predominant component (67.59%), although multiple minor components (glycerol 1,2-diacetate, cinnamyl acetate, caryophyllene oxide, bornyl acetate, terpinolene, α-terpineol, and α-thujene) are also present (Tung et al. 2010; Plata-Rueda et al. 2018). Further, cinnamaldehyde showed more fumigant and contact action against house dust mites than the other EO components (Wang et al. 2011). In addition, cinnamaldehyde is effective for cotton mealy bug pest control but does not negatively impact their natural predators (Abd-Allah and Youssef 2020). Due to their insect integument penetration, other phenylpropanoid compounds (acids, ketones, and esters) were found to have high contact activity against *Sitophilus zeamais* (Zaio et al. 2018).

The presented GC–MS analyses showed that basil EO is rich in linalool (20.07%), trans-α-bergamotene (10.63%), eucalyptol (8.80%), and eugenol (8.62%). Dris et al. (2017) reported that basil EO contains 38 components with two major compounds, linalool (22.52%) and linalyl acetate (53.89%). On the other hand, linalool (35.7%), methyl chavicol (16.3%), trans-α-bergamotene (7.8%), and 1,8-cineole (7.2%) were the basal EO compositions reported in a different study (Giatropoulos et al. 2018). These differences in the components of basil EOs can be attributed to genetic variables, agroclimatic circumstances, and plant morphological variety (Anwar et al. 2021). In our investigation, adulticidal and repulsive effects of basil EO were observed against adult *C. pipiens*. Additionally, adults of *Sitophilus oryzae* and *Tribolium castaneum*, as well as adult *Aedes aegypti*, were repelled by basil EO (Mishra et al. 2012; Kumar et al. 2017). Additionally, adults of *C. pipiens* have been shown to be poisonous and repellent to basil (and eucalyptus) smoke (Osman et al. 2020). Linalool and oleic acids extracted from Melia azedarach showed a high repellency effect against *S. littoralis* larvae (Farag et al. 2011).

Eucalyptus EO is rich in monoterpenoid and phenylpropanoid compounds. Eleven compounds were detected in our GC–MS profile including eucalyptol (49.34%), o-cymene (17.78%), (1R)-2,6,6-trimethylbicyclo[3.1.1]hept-2-ene (13.18%), and terpinene (12.51%). These results are consistent with a previous study that reported oxygenated sesquiterpenes, sesquiterpenes, oxygenated monoterpenes, and monoterpenes in eucalyptus EO (Joshi et al. 2016). Eucalyptol (1, 8-cineol) is a monoterpenoid with high ovipositional deterrent activity and mild feeding repellency for adult mosquitoes (Navayan et al. 2017). Eucalyptus EO is rich in estragole (methyl chavicol,* p*-allylanisole) and a phenylpropene that showed antifeedant and oviposition deterrent effects against housefly and larvicidal activities against mosquitoes (Senthoorraja et al. 2021; Chan et al. 2022). Overall, EO-derived monoterpenes (thujone and linalool) have been reported to be toxic in many insects due to acetylcholinesterase inhibition but are non-toxic to mammals and have low environmental persistence (Cotchakaew and Soonwera 2018). Methyl eugenol was an effective oviposition deterrent in *Phthorimaea operculella* (Wu et al. 2020). The chemical compounds in eucalyptus EO that are responsible for the adulticidal, repellency, and oviposition deterrence in *C. pipiens* are consistent with a previous report that showed that leaf oils from *Eucalyptus citriodora* and *Cinnamomum* species have adulticidal activities in *C. pipiens* (Baz et al. 2022). Previous results showed that *Mentha* species of EOs showed remarkable repellent efficiency and oviposition deterrent activities against *Ae. aegypti* adults (Warikoo et al. 2011; Manh and Tuyet 2020). It has been suggested that the high monoterpenoid content ( +)-menthol, 34.09%; cyclohexanone,5-methyl-2-(1-methylethyl)-,cis, 17.91%; l-menthone, 10.71%; and ( +)-menthylacetat, 9.48%) in peppermint EO like drives the activities observed.

## Conclusion

Mosquito-borne diseases may be mitigated by the use of either adulticidal chemicals that directly impact populations or repellents that reduce olfactory activities that lead to mosquito feeding and oviposition disruption. In this study, cinnamon EO exhibited effective adulticidal, repellence, and oviposition deterrence activities against both laboratory and field-based populations of *C. pipiens*. This strong activity is likely attributable to the high cinnamaldehyde (67.59%) content. Although not as compelling as cinnamon EO, the efficacy of the other three EOs tested for adult mosquito control programs as adulticides, repellents, and oviposition deterrents was sufficient, albeit with decreasing levels of effectiveness (basil > eucalyptus > peppermint). Moreover, GC–MS analysis revealed the composition of the EOs and provided a chemical basis for the observed biological effects of the EOs. Consequently, these EOs are recommended.

### Supplementary Information

Below is the link to the electronic supplementary material.

Supplementary file1 (DOCX 455 KB)

## Data Availability

All data of the study have been presented in the manuscript and the materials which are used in this study are highly quality and grade.
