# Supplementary material for: Chemical composition of four essential oils and their adulticidal, repellence, and field oviposition deterrence activities against Culex pipiens L. (Diptera: Culicidae)
Source: Parasitol Res. 2024 Jan 25;123(1):110. doi: 10.1007/s00436-024-08118-z (PMC10808171; doi:10.1007/s00436-024-08118-z)
Supplement: Supplementary file 1 — Supplementary file1 (DOCX 455 KB) [file 436_2024_8118_MOESM1_ESM.docx]

**Fig S0.**

GC-Mass chromatogram of cinnamon (*Cinnamomum verum*), basil (*Ocimum basilicum*), Tasmanian blue gum (*Eucalyptus globulus*), and peppermint (*Mentha piperita*) essential oils.

Cinnamon (*Cinnamomum verum*)

RT: 0.00 - 45.59 SM: 15B

100

90

80

11.53

NL: 1.55E9

TIC MS

C_Krfa_Dr Motaz

70

Relative Abundance

60

50

40 13.34

30

20

10

3.03

0

7.68

10.01

16.98 18.81

20.88

22.63

25.31 27.80

29.60

32.24

34.23

36.75

42.57 44.38

0 2 4 6 8 10 12 14 16 18 20 22 24 26 28 30 32 34 36 38 40 42 44

Time (min)

Basil (*Ocimum basilicum*)

RT: 0.00 - 45.61 SM: 15B

100

90

80

70

Relative Abundance

60

5.45

7.05 15.38

13.41

17.30 20.30

NL: 1.02E9

TIC MS

B_Rehan_D rMotaz

50

40

30

20 4.40

10

0

8.10

9.40 11.58

25.57

28.59

31.63 34.84

36.90

41.22

0 2 4 6 8 10 12 14 16 18 20 22 24 26 28 30 32 34 36 38 40 42 44

Time (min)

Tasmanian blue gum (*Eucalyptus globulus)*

RT: 0.00 - 45.59 SM: 15B

100

90

80

5.44

NL: 1.16E9

TIC MS

E_Kafor_Dr Motaz

70

Relative Abundance

60

50

40

30

20

10

9.38

0

11.82

15.04

16.99 18.82

20.88

23.77

27.80 29.60 31.54

33.83

35.98

38.01

40.73

0 2 4 6 8 10 12 14 16 18 20 22 24 26 28 30 32 34 36 38 40 42 44

Time (min)

Peppermint (*Mentha piperita*)

RT: 0.00 - 45.59 SM: 15B

100

90

80

9.01

NL: 1.26E9

TIC MS

P_Mint_Dr Motaz

70

Relative Abundance

60

5.45

50

11.76

40

30

20 10.43

10 4.40 15.02

6.99 16.52 18.88

0

20.88

23.76

26.94

30.46

32.87

35.39

37.75

0 2 4 6 8 10 12 14 16 18 20 22 24 26 28 30 32 34 36 38 40 42 44

Time (min)
